# Recurrence of atrial fibrillation after pulmonary vein isolation in dependence of arterial stiffness

**DOI:** 10.1007/s12471-021-01644-w

**Published:** 2021-11-24

**Authors:** T. Shchetynska-Marinova, M. Kranert, S. Baumann, V. Liebe, A. Grafen, S. Gerhards, S. Rosenkaimer, I. Akin, M. Borggrefe, A. L. Hohneck

**Affiliations:** 1 First Department of Medicine—Cardiology, University Medical Centre Mannheim, Medical Faculty Mannheim, University of Heidelberg, Mannheim, Germany; 2 partner site Mannheim, German Centre for Cardiovascular Research (DZHK), Mannheim, Germany

**Keywords:** Atrial fibrillation, Recurrence, Arterial stiffness, Left atrial volume index

## Abstract

**Background:**

Arterial stiffness (AS) has emerged as a strong predictor of cardiovascular (CV) diseases. Although increased AS has been described as a predictor of atrial fibrillation (AF), its role as a risk marker for AF recurrence has not yet been elucidated.

**Methods:**

Patients with AF who underwent pulmonary vein isolation (PVI) were included in this study. Presence of AS was evaluated by measuring aortic distensibility (AD) of the descending aorta by transoesophageal echocardiography.

**Results:**

In total, 151 patients (mean ± standard deviation (SD) age 71.9 ± 9.8 years) were enrolled and followed for a median duration of 21 months (interquartile range 15.0–31.0). During follow-up, AF recurred in 94 (62.3%) patients. AF recurrence was seen more frequently in patients with permanent AF (27% vs 46%, *p* = 0.03) and in those who had undergone prior PVI (9% vs 23%, *p* = 0.02). AD was significantly reduced in patients with AF recurrence (mean ± SD 2.6 ± 2.3 vs 1.5 ± 0.7 × 10^−3^ mm Hg^−1^, *p* < 0.0001), as well as left atrial volume index (LAVI) (mean ± SD 29 ± 12 vs 44 ± 15 ml/m^2^, *p* < 0.0001). Multivariable analysis revealed LAVI (odds ratio (OR) 2.9, 95% confidence interval (CI) 1.2–3.4) and AS (OR 3.6, 95% CI 2.8–4.1) as independent risk factors of AF recurrence.

**Conclusion:**

Increased AS and left atrial size were independent predictors of AF recurrence after PVI. AD as surrogate marker of AS seemed to reflect the overall CV risk. In addition, AD was significantly correlated with left atrial size, which suggests that increased AS leads to atrial remodelling and thus to AF recurrence.

**Trial registration:**

German registry for clinical studies (DRKS), DRKS00019007.

**Supplementary Information:**

The online version of this article (10.1007/s12471-021-01644-w) contains supplementary material, which is available to authorized users.

## What’s new?

- In this study, arterial stiffness was not only a global parameter of cardiovascular damage, but it was also independently associated with recurrent atrial fibrillation.
- Aortic distensibility as a proxy for arterial stiffness was significantly correlated with left atrial size.

## Introduction

Atrial fibrillation (AF) is the most commonly sustained cardiac rhythm disturbance. The prevalence of this age-related condition is still increasing [1, 2]. Due to the large number of patients suffering from AF and given the limited treatment options, early detection and prevention are becoming increasingly important [3]. Although the treatment options for AF are still improving, especially interventional therapy by catheter ablation or pulmonary vein isolation (PVI), recurrences remain a major challenge.

Several predictors of recurrent AF have been identified, such as persistent AF, left atrial (LA) size or chronic kidney disease [4–7]. These predictors are also included in the APPLE score [8], a novel score for prediction of rhythm outcome. Most factors included in the APPLE score are cardiovascular (CV) components that are associated with increased AS [9]. Arterial stiffness (AS) also increases with age and is an independent predictor of CV diseases and mortality [10]. Furthermore, an association between AS and an increased risk of new-onset AF has recently been described [11, 12].

Since AS provides information on the overall CV risk, as well as on subclinical organ damage, noninvasive methods for the assessment of AS are becoming increasingly important in prevention and risk stratification [13].

Measurement of aortic distensibility (AD) as surrogate of AS, by using transoesophageal echocardiography, permits a convenient assessment, which can be easily incorporated into clinical routine of patients with AF [14]. This method could not only be time effective but is also potentially superior to conventional risk stratification. We therefore investigated the role of AD, as a surrogate of increased AS and overall CV risk, in the prediction of recurrence of AF after PVI.

## Methods

### Study population

This monocentric observational trial was conducted at the First Department of Medicine (Cardiology), University Medical Centre Mannheim in Mannheim, Germany. Patients with AF who underwent PVI were included in the study. Written informed consent was obtained from all patients. Recruitment started in June 2015 and ended in December 2017. Patients were followed prospectively. Other results of this study population have been reported previously [6].

Baseline demographic and clinical characteristics were acquired from a questionnaire and patient charts. Exclusion criteria at the time of transoesophageal echocardiography were acute myocardial infarction or stroke (within past 30 days), indication for aortocoronary bypass operation, minimal heart rate at rest <50 bpm, need for pacemaker stimulation, hypotension with blood pressure <90/50 mm Hg or uncontrolled hypertension with systolic blood pressure ≥160 mm Hg, history of aortic dissection/aneurysm and history of aortic prosthesis.

The study was conducted according to the principles of the Declaration of Helsinki and was approved by the local ethics committee, the Medical Ethics Commission II, Faculty of Medicine Mannheim, University of Heidelberg, Germany. Data were analysed anonymously. Data protection was in accordance with the European Union’s Data Protection Directive.

### Assessment of atrial fibrillation

Recurrence of AF was based on electrocardiographic evidence, including 72-hour Holter monitoring. Classification of AF was performed in accordance with the current European Society of Cardiology (ESC) guidelines for the management of atrial fibrillation [15], whereby two patterns—paroxysmal and persistent AF—were distinguished.

Prior to PVI, clinical risk scores for prediction of thromboembolism (CHA_2_DS_2_-VASc score: congestive heart failure or left ventricular systolic dysfunction, hypertension, age ≥75 years, diabetes mellitus, prior stroke or transient ischaemic attack, vascular disease, age 65–74 years, female sex [16]), rhythm outcome after ablation (APPLE score: age >65 years, persistent AF, impaired estimated glomerular filtration rate (eGFR) <60 ml/min per 1.73 m^2^, LA diameter ≥43 mm, ejection fraction <50% [8]) and AF symptom classification (European Heart Rhythm Association score [17]) were assessed.

### Echocardiography

Transthoracic and transoesophageal echocardiographic examinations were performed at the time of study inclusion using Vivid E9 (GE Vingmed Ultrasound, Horten, Norway) or iE33 (Philips Medical Systems, Andover, MA, USA).

Cardiac chamber size and function were assessed using standard M‑mode, 2‑dimensional (2D) and colour Doppler imaging [18]. Left ventricular ejection fraction (LVEF) was calculated using Simpson’s method. LA measurements were performed at end-systole in the apical two-chamber (2Ch) and four-chamber (4Ch) views. Echocardiographic measurements of diastolic function were performed according to the recommendation for the evaluation of LV diastolic function by echocardiography [19].

The descending aorta was routinely imaged at the end of each assessment. Cross-sectional systolic and diastolic aortic diameters were measured with an electrocardiogram as a guide using 2D imaging techniques [20]. If echocardiography was performed for AF, measurements from the available heartbeats were averaged (usually over 3 to 5 cardiac cycles). If available, measurements were performed at multiple levels of the descending aorta. Brachial blood pressures were measured at the time of transoesophageal echocardiography using standardised protocols.

AD was calculated using the aortic diameters and brachial blood pressures as follows [21]:

$$\mathrm{AD}=(\text{AoSD}-\text{AoDD})/\text{AoDD}\times (\mathrm{SBP}-\mathrm{DBP})$$

*AoSD* aortic systolic diameter, *AoDD* aortic diastolic diameter, *SBP* systolic blood pressure, *DBP* diastolic blood pressure.

### Reproducibility

To ensure reproducibility of AD assessment, a randomly generated set of 40 transoesophageal echocardiographic scans was analysed before study enrolment by one single experienced observer (TS-M), who was blinded to the data. Intraobserver variability for AD was determined using the calculation of intraclass correlation coefficient (ICC).

### Pulmonary vein isolation

A diagnostic quadripolar electrode catheter (EP Technologies Inc., Sunnyvale, CA, USA) was positioned in the coronary sinus with a femoral vein approach. Transseptal catheterisation was performed, and systemic anticoagulation was achieved with intravenous heparin to maintain an activated clotting time of 250–350 s.

A multipolar Lasso catheter (Biosense Webster Inc., Irvine, CA, USA) was placed into the left atrium to map signals at the ostial sides of the pulmonary veins. A deflectable, quadripolar 7‑Fr catheter with 2‑5-2-mm interelectrode spacing and a 4-mm distal electrode with an embedded thermistor (EP Technologies Inc., Sunnyvale, CA, USA) was inserted into the left atrium, either through the same transseptal puncture site or through a second transseptal puncture, and was used for ablation. Bipolar and unipolar electrograms were filtered at bandpass settings of 30–500 Hz and 0.05–200 Hz, respectively, and were recorded digitally (EPMed Systems Inc., Mount Arlington, NJ, USA).

Pacing was performed from the coronary sinus or left atrium with a stimulator (Model EP‑3 Clinical Stimulator, EPMed Systems Inc., Mount Arlington, NJ, USA). Complete isolation was verified as a reduction of all signals ≤0.2 mV and by pacing manoeuvres.

### Primary endpoint

Recurrence of AF after PVI, based on electrocardiographic evidence, served as primary endpoint.

### Follow-up

Patients with AF were followed up for a median duration (interquartile, IQR) of 21 months (15.0–31.0). Periodical follow-up was performed during routine visits in our outpatient clinic. Three months after PVI, 72-hour Holter monitoring was performed.

### Statistical analysis

Data are presented as mean ± standard deviation, median (IQR) or frequency (percentage). Continuous variables were compared using a two-tailed Student’s *t*-test for parametric and Mann-Whitney U test for nonparametric variables. Categorical variables were compared with a *χ*^2^ test.

Correlation analysis was performed with univariate linear regression analysis to test associations between AD as dependent variable and several clinical characteristics. Multivariable analysis with logistic regression analysis was performed using block entry of variables with *p* < 0.01 in univariate analysis. There variables were: eGFR (ml/min per 1.73 m^2^), heart failure, treatment with amiodarone, CHA_2_DS_2_-VASc score, APPLE score, left atrial volume index (LAVI) (in ml/m^2^) and AD. ICC was calculated to assess intraobserver variability for AD, providing a coefficient ranging from 0 to 1 and its 95% confidence interval (CI), with an ICC close to 1 indicating high similarity.

For all statistical analyses, *p* < 0.05 was considered statistically significant. All statistical analyses were performed with Statistical 1 Package for Social Sciences (SPSS for Windows 24.0, Chicago, IL, USA) or GraphPad Prism 8.0 (GraphPad Software Inc., San Diego, CA, USA).

## Results

### Baseline

In total, 151 patients were included in the study (mean age 71.9 ± 9.8 years, 64% male). The patients suffered from several cardiovascular comorbidities, such as hypertension (78%), diabetes mellitus (13%), chronic kidney disease (32%), coronary artery disease (29%), heart failure (43%) and obstructive sleep apnoea (13%). Stroke or transitory ischaemic attack had occurred in 12% of the patients and 18% had a history of smoking.

Concomitant medical treatment was in accordance with the 2016 ESC Guidelines for the management and treatment of atrial fibrillation [15]; 72% of the patients were taking a betablocker, 54% an angiotensin-converting enzyme inhibitor or angiotensin receptor blocker and 19% a calcium channel blocker. Statin use was observed in 41% of the patients and 7% received diabetes medication. A small proportion was on antiarrhythmic treatment with amiodarone (11%) or digitalis (9%).

Persistent AF was diagnosed in 59 patients (39%). Mean CHA_2_DS_2_-VASc score was 3.0 ± 1.7, while mean APPLE score was 1.9 ± 1.2. Prior PVI had been performed in 27 patients (18%). Hundred and thirty-five patients (89%) received anticoagulant therapy, consisting of vitamin K antagonists or direct oral anticoagulants.

Most patients had a normal systolic cardiac function (LVEF 56 ± 9%); impaired LVEF (<50%) was found in 25 patients (27%). Mean LA dimensions, both LA diameter measured by M‑mode (42 ± 7 mm) and LAVI (40 ± 16 ml/m^2^) were elevated and outside of the normal range [18, 22]. Mean AD was reduced (1.9 ± 1.1 × 10^−3^ mm Hg^−1^) compared with normal values [23].

Demographics, baseline characteristics and drug treatment specifics of the study population are shown in Tab. S1 in the Electronic Supplementary Material.

### Follow-up

During follow-up, AF recurred in 94 patients (62.3%). We subsequently divided the cohort into two groups: patients without or with recurrent AF. Vital parameters (heart rate, blood pressure and pulse pressure) were not different between the groups. Patients with recurrent AF more often had hypertension (66% vs 83%, *p* = 0.04), heart failure (24% vs 54%, *p* < 0.0001) and a higher CHA_2_DS_2_-VASc score (2.5 ± 1.7 vs 3.3 ± 1.6, *p* = 0.004). Chronic kidney disease was also more common in patients with AF recurrence (17.5% vs 40.4%, *p* = 0.003), with significantly lower eGFR values (62 ± 14 vs 56 ± 14 ml/min per 1.73 m^2^, *p* = 0.009). The presence of CV risk factors, such as nicotine consumption or diabetes, was similar. There were no differences regarding concomitant treatment with antihypertensive drugs, statins, diabetes medication or anticoagulant therapy. In contrast, treatment with amiodarone (1.8% vs 16.0%, *p* < 0.01) or digitalis (1.8% vs 13.8%, *p* = 0.02) was more common in the recurrence group. AF recurred more often in patients with persistent AF (27% vs 46%, *p* = 0.03) and in those who had undergone prior PVI (9% vs 23%, *p* = 0.02). Patients with recurrent AF had a significantly higher APPLE score (1.4 ± 1.0 vs 2.2 ± 1.2, *p* < 0.0001).

**Fig. 1:**
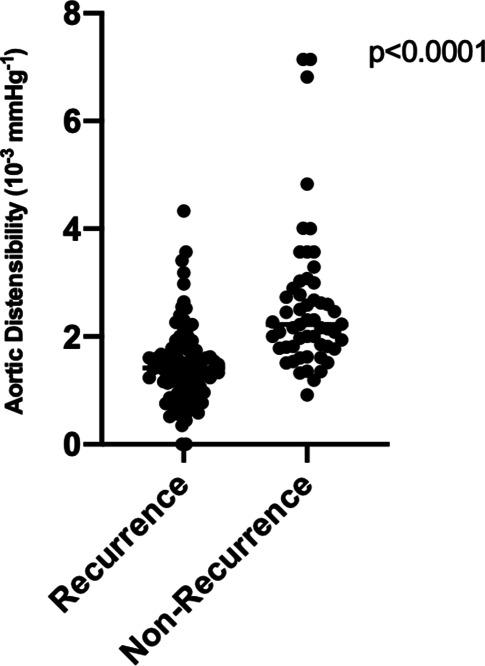
Recurrence of atrial fibrillation in dependence of aortic distensibility

LVEF was lower in patients with AF recurrence (58 ± 7 vs 54 ± 10%, *p* = 0.02), with a higher proportion of patients with impaired LVEF in the recurrence group (9% vs 21%, *p* = 0.04). LA dimensions and volumes were significantly higher in patients with recurrent AF (LA M‑mode 40 ± 6 vs 43 ± 7 mm, *p* = 0.01; LAVI 29 ± 12 vs 44 ± 15 ml/m^2^, *p* < 0.0001). AD was significantly reduced in patients with AF recurrence (2.6 ± 2.3 vs 1.5 ± 0.7 × 10^−3^ mm Hg^−1^, *p* < 0.0001) (Fig. 1). ICC was calculated for the assessment of intraobserver variability of AD and showed excellent to nearly perfect agreement (0.92, 0.81–0.96). Complete results are displayed in Tab. S1 (see Electronic Supplementary Material).

Variables with *p* < 0.01 in univariate analysis were then studied in multivariable analysis, which revealed LAVI (odds ratio (OR) 2.9, 95% CI 1.2–3.4) and AS (OR 3.6, 95% CI 2.8–4.1) were independent risk factors for AF recurrence. The other variables failed to reach statistical significance (Tab. S2 in the Electronic Supplementary Material).

To determine the potential significance of AD as a surrogate of overall CV risk, correlation analysis was performed, with AD as the dependent variable. AD was significantly correlated with age (r = −0.23, *p* = 0.005), hypertension (r = −0.27, *p* = 0.0001), as well as systolic blood pressure (r = −0.19, *p* = 0.02) and pulse pressure (r = −0.24, *p* = 0.004), eGFR (r = 0.31, *p* = 0.0001), heart failure (r = −0.26, *p* = 0.002) and CHA_2_DS_2_-VASc score (r = −0.25, *p* = 0.002). Coronary three-vessel disease (r = −0.17, *p* = 0.03), use of vitamin K antagonists (r = −0.18, *p* = 0.03) and LVEF (r = 0.18, *p* = 0.03) showed less strong associations. In addition, a significant inverse correlation was seen for LA dimensions (LA M‑mode, r = −0.22, *p* = 0.04) and LA volumes (LA volume 4Ch: r = −0.31, *p* = 0.002; LAVI: r = −0.36, *p* = 0.0003) (Tab. 1).

**Table 1:** Correlation analysis of AD and clinical characteristics

Variable	r (95% CI)	*P*-value
Age, years	−0.23 (−0.43 to −0.13)	0.005
Systolic blood pressure, mm Hg	−0.19 (−0.34 to −0.02)	0.02
Pulse pressure, mm Hg	−0.24 (−0.38 to −0.08)	0.004
Hypertension	−0.27 (−0.41 to −0.11)	0.001
eGFR, ml/min per 1.73 m^2^	0.31 (0.16 to 0.45)	0.0001
Three-vessel disease	−0.17 (−0.33 to −0.01)	0.03
Heart failure	−0.26 (−0.41 to −0.09)	0.002
CHA_2_DS_2_-VASc score	−0.25 (−0.40 to −0.09)	0.002
VKA use	−0.18 (−0.33 to −0.01)	0.03
LVEF, %	0.18 (0.02 to 0.34)	0.03
LA M‑mode, mm	−0.22 (−0.41 to −0.01)	0.04
LA volume 4Ch, ml	−0.31 (−0.47 to −0.11)	0.002
LAVI, ml/m^2^	−0.36 (−0.52 to −0.17)	0.0003

*CI* confidence interval, *eGFR* estimated glomerular filtration rate, *VKA* vitamin K antagonist, *LVEF* left ventricular ejection fraction, *LA* left atrial, *4Ch* four-chamber view, *LAVI* left atrial volume index

## Discussion

In the present study, we explored the predictive role of AS in AF recurrence after PVI. The results of our analysis showed that patients with decreased AD were more likely to have recurrences of AF. Besides AD, LAVI remained an independent predictor after multivariable analysis.

In recent years, several studies have demonstrated the impact of various clinical parameters to predict AF recurrence. Generally, it is recognised that permanent AF, LA size, hypertension, diabetes mellitus, obstructive sleep apnoea, heart failure, and early AF recurrence after PVI [24–29] are associated with recurrent AF, which is why multimorbid patients are more likely to be considered at risk of recurrence.

AF type and severity of symptoms are the most well-established parameters when selecting a rhythm control strategy [30]. There is strong evidence showing that persistent AF is associated with a higher recurrence rate and a higher stroke and mortality risk than paroxysmal AF [30]. Our data support these previously reported findings, with an increase in AF recurrence in patients with persistent AF compared with those with paroxysmal AF. However, in our study, the AF phenotype lost its predictive value when adjusting for other predictors in multivariable analysis. A possible explanation is that not the AF phenotype itself but the underlying comorbidities and possibly the level of atrial fibrosis and inflammation, as shown by Marrouche et al., are the main risk factors for recurrences of AF [31].

In recent years, AS has emerged as a strong predictor of CV disease, end-organ damage and all-cause mortality. Moreover, several clinical studies have reported that AS is associated with new-onset AF [32–34], as well as with AF recurrence after initially successful restoration of sinus rhythm [35–38]. It is recognised that the stiffening of large arteries and the aorta may exert severe effects on CV function, including cardiac remodelling and diastolic dysfunction, systolic hypertension, and reduced coronary perfusion [39, 40]. Furthermore, excessive pressure pulsatility may contribute to microvascular dysfunction, leading to end-organ damage [39]. Given that increased AS may be present long before the first clinical manifestations of adverse haemodynamics and end-organ damage appear [41, 42], AS can be used as a valid indicator of the likelihood of success of attempts at rhythm control and thus influence clinical decision-making to choose the best treatment option in patients with AF. Especially in patients who are considered to have ‘a low risk of recurrence’, due to the absence of conventional risk factors, measuring AD could provide earlier information on structural alterations that are also associated with an increased risk of recurrence.

In our study, AD was significantly reduced in patients with recurrent AF, reflecting increased AS. Few previous reports, which used the cardio-ankle vascular index and peripheral pulse wave velocity as surrogate measures of arterial stiffness, enforce our results [36, 43, 44]. Fumagalli et al. demonstrated that the presence of AF during follow-up is directly correlated with AS, as assessed by the cardio-ankle vascular index [44]. Lau et al. found that patients with the highest levels (≥75th percentile) of peripheral pulse pressure, central pulse pressure and augmentation pressure have higher recurrence rates of lone AF [36]. In contrast, Kizilirmak et al. did not find an independent association between parameters of AS and AF recurrence and concluded that LA size is the strongest independent predictor of AF recurrence [37]. Although the matter remains controversial, which may also be due to the lack of standardisation in the assessment of AS, the preposition that AS can be used as a valid predictor of AF recurrence is pathophysiologically plausible.

Several mechanisms may explain the association between AS and AF. Increased AS and its pathological haemodynamic pattern may be associated with increased afterload, decreased LV compliance, and subsequent LA remodelling and enlargement, which may represent a substrate for development maintenance of AF [45]. Furthermore, increased AS results in excessive pulsatility in the aorta, which is transmitted preferentially to low-resistance vascular beds (such as the kidney, liver and brain) and causes end-organ damage. These events may worsen the comorbidity state in AF patients, which could also contribute to AF progression. Thus, considering the adverse haemodynamic effects of AS, its predictive role in AF recurrence is not surprising.

Interestingly, the possible consequences of increased AS discussed above (chronic kidney disease, hypertension, heart failure) were also significantly associated with recurrences of AF in our study. However, it cannot be ascertained whether increased AS is an independent risk factor for the progression of AF or a global marker of overall CV risk and end-organ dysfunction. On the other hand, AF itself could promote negative vascular changes by promoting atherosclerosis and inflammation. Therefore, we believe that both increased AS and AF could promote each other, forming a vicious circle of adverse haemodynamic, structural and functional CV changes.

Since the LA diameter does not reflect the correct size of the left atrium, measuring LA volume is a more correct approach [46]. For this reason, LAVI has been increasingly used as a proxy for LA size in clinical trials. The risk of AF recurrence increases with the size of the left atrium [47], whereby a cut-off value >34 ml/m^2^ is seen in several studies [6, 48]. In the current study, LAVI was identified as an independent predictor of AF recurrence. Furthermore, we found a significant correlation of LAVI with AD. This is in accordance with the literature.

In hypertensive patients, AS is significantly correlated with LA size [49], which leads to the assumption that AS is not only a surrogate for CV risk but directly promotes structural cardiac changes [50]. These adverse effects appear to be reversible, to a certain extent. This has been demonstrated by Tsang et al., who showed that treatment with quinapril leads to a decrease in LAVI and an improvement in AS [51]. AS could therefore be the missing link, leading to recurrent AF through atrial remodelling.

For this reason, AS could be an interesting therapeutic target to break the vicious circle and to help identify patients at higher risk. AD, in contrast to LAVI, could be an early marker because increased LAVI indicates the terminal stage of cardiac remodelling, while AS reflects an ongoing process. On the other hand, AD is not a specific parameter and is influenced by various factors. In our cohort, AD was significantly correlated with age, blood pressure, kidney function, heart failure and LA size. Scoring systems such as the CHA_2_DS_2_-VASc score and APPLE score are composed of many of these parameters, which are used to assess the risk of AF recurrence. The implementation of AD in risk stratification of patients with AF could thus provide a simplified approach to reduce these complex scoring systems to a single value. Since AS may predict the risk of AF recurrence independently of traditional risk factors and is also likely to predict overall CV risk, its implementation in clinical practice could lead to both time savings and better prediction. Compared with established factors such as LAVI, AD was not inferior.

### Study limitations

Our study population consisted of patients with complicated AF who were treated at a university hospital and not by a general physician, which may have led to a higher proportion of AF recurrences. On the other hand, especially in asymptomatic patients, the incidence of recurrent AF may have been underestimated. Even though Holter monitoring was performed periodically, paroxysmal episodes may not have been detected.

## Conclusion

The current study evaluated the predictive role of AS in AF recurrence. Both AS, measured as AD, and LA size were independently positively associated with AF recurrence during follow-up. In addition, AD was significantly correlated with LA size, which leads to the assumption that increased AS promotes cardiac remodelling and hence maintains AF. For this reason, the use of AD measurements may help in the evaluation of the individual risk of AF recurrence after rhythm control therapy. However, further randomised clinical trials are needed to prove whether risk prediction by evaluating AD is not only time effective but also more accurate than conventional risk stratification. Our results should be verified in a larger and less complex patient population, in order to derive a recommendation for clinical practice.

## Supplementary Information

Table S1 Patients’ demographics, baseline characteristics, concomitant diseases and drug treatment

Table S2 Performance of a multivariable analysis to determine variables independently associated with an increased risk of atrial fibrillation recurrence during follow-up
