# Supplementary material for: Recurrence of atrial fibrillation after pulmonary vein isolation in dependence of arterial stiffness
Source: Neth Heart J. 2021 Nov 24;30(4):198–206. doi: 10.1007/s12471-021-01644-w (PMC8941046; doi:10.1007/s12471-021-01644-w)
Supplement: Supplementary file 1 — Table S1 Patients’ demographics, baseline characteristics, concomitant diseases and drug treatment [file 12471_2021_1644_MOESM1_ESM.docx]

**Tab. S1** Patients’ demographics, baseline characteristics, concomitant diseases and drug treatment

|  | All patients with AF (n=151) | Without recurrent AF (n=57, 37.8%) | Recurrent AF (n=94, 62.3%) | *p*-value |
| --- | --- | --- | --- | --- |
| Age (years) | 71.9 ± 9.8 | 70.1 ± 10.1 | 73.0 ± 9.5 | 0.07 |
| Sex, male (%) | 96 (63.6) | 41 (71.9) | 55 (58.5) | 0.10 |
| BMI (kg/m^2^) | 27.8 ± 5.5 | 28.5 ± 5.8 | 27.3 ± 5.3 | 0.22 |
| **Cardiovascular parameters** | | | | |
| Heart rate (bpm) | 70.2 ± 14.2 | 70.0 ± 12.2 | 70.5 ± 15.4 | 0.81 |
| SBP (mmHg) | 128.9 ± 16.3 | 126.4 ± 15.7 | 130.3 ±16.6 | 0.15 |
| DBP (mmHg) | 76.8 ± 9.1 | 76.4 ± 8.9 | 77.1 ± 9.2 | 0.63 |
| PP (mmHg) | 52.0 ± 13.7 | 50.0 ± 12.1 | 53.2 ± 14.5 | 0.16 |
| **Concomitant diseases, n (%)** | | | | |
| Hypertension | 117 (77.5) | 39 (66.1) | 78 (83.0) | **0.04** |
| Diabetes | 19 (12.6) | 4 (6.8) | 15 (16.0) | 0.11 |
| History of smoking | 27 (17.9) | 11 (18.6) | 16 (17.0) | 0.73 |
| CKD (GFR < 60) | 48 (31.8) | 10 (17.5) | 38 (40.4) | **0.003** |
| Creatinine (mg/dl) | 1.13 ± 0.52 | 1.04 ± 0.25 | 1.18 ± 0.63 | 0.12 |
| GFR (ml/min/1.73m^2^) | 58.6 ± 13.3 | 62.2 ± 13.5 | 56.4 ± 12.8 | **0.009** |
| Prior stroke/ TIA | 18 (11.9) | 7 (11.9) | 11 (11.7) | 0.92 |
| CAD | 44 (29.1) | 12 (20.3) | 32 (34.0) | 0.09 |
| 3-vessel disease | 14 (9.3) | 3 (5.1) | 11 (11.7) | 0.19 |
| Heart failure | 65 (43.1) | 14 (23.7) | 51 (54.3) | **< 0.0001** |
| ≥ moderate MI | 21 (13.9) | 7 (11.9) | 14 (14.9) | 0.66 |
| ≥ moderate MS | 5 (3.3) | 3 (5.1) | 2 (2.1) | 0.31 |
| Obstructive sleep apnea | 20 (13.3) | 10 (10.6) | 10 (17.0) | 0.23 |
| **Concomitant medication, n (%)** | | | | |
| Betablocker | 109 (72.2) | 37 (64.9) | 72 (78.3) | 0.22 |
| Calcium channel blocker | 29 (19.2) | 12 (21.1) | 17 (18.5) | 0.58 |
| ACE inhibitor/ ARB | 82 (54.3) | 30 (52.6) | 52 (56.5) | 0.93 |
| Amiodarone | 16 (10.6) | 1 (1.8) | 15 (16.0) | **0.007** |
| Digitalis | 14 (9.3) | 1 (1.8) | 13 (13.8) | **0.02** |
| Statin | 62 (41.1) | 19 (33.3) | 43 (45.7) | 0.21 |
| Diabetes medication | 11 (7.3) | 2 (3.5) | 9 (9.8) | 0.18 |
| **AF characteristics** | | | | |
| Persistent AF | 59 (39.1) | 16 (27.1) | 43 (45.7) | **0.03** |
| CHA_2_DS_2_-VASc score | 3.0 ± 1.7 | 2.5 ± 1.7 | 3.3 ± 1.6 | **0.004** |
| EHRA score | 2.3 ± 0.9 | 2.0 ± 0.8 | 2.4 ± 0.9 | **0.02** |
| APPLE score | 1.9 ± 1.2 | 1.4 ± 1.0 | 2.2 ± 1.2 | **< 0.0001** |
| Prior PVI | 27 (17.9) | 5 (8.8) | 22 (23.4) | **0.02** |
| **Anticoagulant therapy, n (%)** | | | | |
| VKA | 11 (7.3) | 2 (3.5) | 9 (9.8) | 0.18 |
| DOAC | 124 (82.1) | 46 (80.7) | 78 (84.8) | 0.92 |
| **Echocardiographic parameters** | | | | |
| LVEF (%) | 55.5 ± 9.3 | 57.7 ± 7.4 | 54.1 ± 10.1 | **0.02** |
| LVEF < 50% | 25 (26.6) | 5 (8.8) | 20 (21.3) | **0.04** |
| LVEDD | 48.4 ± 6.2 | 47.0 ± 6.1 | 49.1 ± 6.2 | 0.13 |
| LA M-Mode (mm) | 41.7 ± 6.6 | 40.0 ± 6.0 | 42.8 ± 6.6 | **0.01** |
| LA-Volume 4Ch (ml) | 75.7 ± 29.5 | 60.0 ± 27.6 | 82.7 ± 27.7 | **< 0.0001** |
| LA Volume index (ml/m2) | 39.5 ± 15.8 | 29.2 ± 11.5 | 44.2 ± 15.3 | **< 0.0001** |
| Aortic distensibility (10^-3^mmHg^-1^) | 1.90 ± 1.11 | 2.58 ± 1.33 | 1.49 ± 0.70 | **< 0.0001** |
| Circumferential strain | 0.08 ± 0.04 | 0.10 ± 0.04 | 0.07 ± 0.03 | **< 0.0001** |

Data are presented as mean ± SD or n (%) of subjects. ACE: Angiotensin converting enzyme; AF: atrial fibrillation; ARB: aldosterone receptor blocker; BMI: body mass index; bpm: beats per minute; CAD: coronary artery disease; DBP: diastolic blood pressure; DOAC: direct oral anticoagulant; EHRA: European Heart Rhythm Association; GFR: glomerular filtration rate; MI: mitral insufficiency; MS: mitral stenosis; NYHA: New York Heart Association; PP: pulse pressure; PVI: pulmonary vein isolation; SBP: systolic blood pressure; TIA: transitoric ischemic attack; VKA: vitamin K antagonist.
