# Supplementary material for: Recurrence of atrial fibrillation after pulmonary vein isolation in dependence of arterial stiffness
Source: Neth Heart J. 2021 Nov 24;30(4):198–206. doi: 10.1007/s12471-021-01644-w (PMC8941046; doi:10.1007/s12471-021-01644-w)
Supplement: Supplementary file 2 — Table S2 Performance of a multivariable analysis to determine variables independently associated with an increased risk of atrial fibrillation recurrence during follow-up [file 12471_2021_1644_MOESM2_ESM.docx]

**Tab. S2** Performance of a multivariable analysis to determine variables independently associated with an increased risk of AF recurrence during follow-up^a^

|  | OR | 95%CI | p-value |
| --- | --- | --- | --- |
| GFR (ml/min/1.73m^2^) | - | - | 0.76 |
| Heart failure | - | - | 0.74 |
| Treatment with amiodarone | - | - | 0.24 |
| CHA2DS2-VASc score | - | - | 0.38 |
| APPLE score | - | - | 0.79 |
| LAVI (ml/m2) | 2.9 | 1.2 to 3.4 | **0.005** |
| Aortic distensibility | 3.6 | 2.8 to 4.1 | **0.0005** |

^a^ Parameters provided to have a p<0.01 in univariate analysis were included as independent variables and AF recurrence as dependent variable

CI, confidence interval; GFR, glomerular filtration index; LAVI, left atrial volume index, OR, odds ratio.
